# Seeing the world through non rose-colored glasses: anxiety and the amygdala response to blended expressions

**DOI:** 10.3389/fnhum.2015.00152

**Published:** 2015-03-27

**Authors:** Sonia J. Bishop, Geoffrey K. Aguirre, Anwar O. Nunez-Elizalde, Daniel Toker

**Affiliations:** ^1^Department of Psychology, University of California BerkeleyBerkeley, CA, USA; ^2^Helen Wills Neuroscience Institute, University of California BerkeleyBerkeley, CA, USA; ^3^Department of Neurology, University of PennsylvaniaPhiladelphia, PA, USA

**Keywords:** fMRI adaptation, expression, amygdala, anxiety, fear, ambiguity, face processing, representation

## Abstract

Anxious individuals have a greater tendency to categorize faces with ambiguous emotional expressions as fearful (Richards et al., 2002). These behavioral findings might reflect anxiety-related biases in stimulus representation within the human amygdala. Here, we used functional magnetic resonance imaging (fMRI) together with a continuous adaptation design to investigate the representation of faces from three expression continua (surprise-fear, sadness-fear, and surprise-sadness) within the amygdala and other brain regions implicated in face processing. Fifty-four healthy adult participants completed a face expression categorization task. Nineteen of these participants also viewed the same expressions presented using type 1 index 1 sequences while fMRI data were acquired. Behavioral analyses revealed an anxiety-related categorization bias in the surprise-fear continuum alone. Here, elevated anxiety was associated with a more rapid transition from surprise to fear responses as a function of percentage fear in the face presented, leading to increased fear categorizations for faces with a mid-way blend of surprise and fear. fMRI analyses revealed that high trait anxious participants also showed greater representational similarity, as indexed by greater adaptation of the Blood Oxygenation Level Dependent (BOLD) signal, between 50/50 surprise/fear expression blends and faces from the fear end of the surprise-fear continuum in both the right amygdala and right fusiform face area (FFA). No equivalent biases were observed for the other expression continua. These findings suggest that anxiety-related biases in the processing of expressions intermediate between surprise and fear may be linked to differential representation of these stimuli in the amygdala and FFA. The absence of anxiety-related biases for the sad-fear continuum might reflect intermediate expressions from the surprise-fear continuum being most ambiguous in threat-relevance.

## Introduction

Facial expressions are important social cues. They signal emotional state and can act as warning signals that threat is imminent. A stranger with a look of fear may indicate a threat that you have not seen, or that they have perceived your behavior as aggression. In either scenario, detecting and evaluating that signal is of clear importance. The amygdala has been proposed to be the neural locus of the rapid processing of potential threat cues including fearful expressions (Vuilleumier et al., 2001; Öhman, 2005).

There can be uncertainty in whether a given stimulus signals the presence of threat. High trait anxious individuals show an increased tendency to interpret ambiguous stimuli as threat-related (Bishop, 2007). Early demonstrations of this used text where both a “threat” and a “neutral” meaning was available (Mathews and MacLeod, 1994). Extending this work, Richards and colleagues examined the categorization of blended emotional expressions created by morphing between exemplars of basic emotions (Richards et al., 2002). They observed that high trait anxious individuals made more fear categorizations than low anxious participants for expression blends that contained 50% or higher proportion of fear, plus 50% or lower of surprise or sadness. Biases were not observed for other negative expression blends (e.g., anger/sadness) or for blends of surprise and happiness. Based on these results, they hypothesized that categorization biases for expressions containing fear might reflect increased amygdala responses to such stimuli in anxious individuals.

The current study examined whether trait anxiety is indeed associated with altered representation in the amygdala of expressions containing fear. A functional magnetic resonance imaging (fMRI) “continuous carry-over” adaptation paradigm (Aguirre, 2007; Harris and Aguirre, 2010) was used to examine the representation of pure (sad, fear, surprise) and blended expressions within the amygdala. The principle behind fMRI adaptation is that if a given brain region codes for a particular stimulus attribute, then a decrease in brain activity, as indexed by the Blood Oxygenation Level Dependent (BOLD) signal, will be observed as a function of whether two sequentially viewed stimuli share this attribute (Grill-Spector et al., 2006). Similarly, “release from adaptation,” as indexed by an increase in BOLD signal, is expected to occur as a function of the extent to which two sequentially viewed stimuli differ in regards to the attribute in question. In the fMRI adaptation design most classically used, each trial involves close sequential presentation of two stimuli, for example two faces. The first stimulus in each pair is kept constant across conditions and the second stimulus is varied. In face adaptation studies, “control” trials typically consist of two identical faces, while in other conditions the second face differs from the first in identity, expression, viewpoint, or another attribute of interest (Winston et al., 2004; Xu and Biederman, 2010). The extent to which BOLD activity is elevated on trials where the second face differs from the first in a given attribute (identity, expression etc.) relative to control trials, where the two faces are identical, gives a measure of release from adaptation as a function of change in that particular attribute, and as such is argued to indicate that the region or voxel examined is sensitive to the attribute in question. The continuous carry-over adaptation paradigm (Aguirre, 2007) differs from the classic approach as follows. Most importantly, stimuli are not presented in pairs but rather as a single long rapid sequence of trials typically separated by no more than a second, with null trials interspersed. A second key feature of the design is that a “type 1 index 1” trial sequence is used—this permutes trial order such that every trial type follows every other trial type (and itself) equally often (Finney and Outhwaite, 1956; Aguirre, 2007). As a result, it is possible to independently model the BOLD activity to each stimulus as a function of not only what it is (the “direct” effect), but also how it differs in terms of a given feature from the stimulus that preceded it (the “adaptation” or “carry-over” effect). This makes it possible to take advantage of, rather than have to control for, prior stimulus effects, and to use an extremely rapid sequence of presentation. By such means, it is possible to examine how adaptation varies as a function of changes along the feature dimension of interest. This in turn facilitates examination of the similarity, or dissimilarity, with which stimuli that differ in some attribute (e.g., percentage of fear in the expression viewed) are represented in a given brain region.

Here, we used a continuous carry over design to test the prediction that high trait anxious participants would show a bias in representation of intermediate expression morphs containing fear. Specifically, we tested the hypothesis that high trait anxious individuals would show greater adaptation of the amygdala BOLD signal—indicative of greater representational similarity—for transitions between 50/50 fear/surprise or fear/sadness morphs and expressions predominantly (66–100%) containing fear than between these 50/50 morphs and expressions predominantly (66–100%) containing surprise or sadness, respectively. In the study by Richards and colleagues, results from fear-surprise and fear-sad continua were collapsed. Hence our secondary aim was to determine whether biases in categorization behavior and fMRI adaptation effects would be observed in both or just one of these continua. Our third aim was to establish whether anxiety-related differences in categorization performance and amygdala adaptation effects would only be observed for expressions containing some percentage of fear or whether differences would also be observed for blends between surprise and sadness.

## Materials and methods

### Participants

A total of 54 healthy adults participated in the study. Nineteen participants (13 female, all right-handed, aged 19–24 years, mean age 20.5 years) completed a combined fMRI and behavioral experimental session. An additional 35 participants (25 females, all right-handed, aged 18–36 years, mean age 20.8 years) completed the behavioral task alone. Individuals with a history of psychiatric care, neurological disease, or head injury were excluded from the study, as were individuals using psychotropic drugs or with a significant history of illegal drug use. The study was approved by the University of California Berkeley committee for protection of human subjects. Informed consent was obtained from all participants prior to participation.

### Procedure

We measured trait anxiety using the Spielberger State-Trait Anxiety Inventory, Form Y (STAI; Spielberger et al., 1983). This self-report questionnaire provides a measure of trait vulnerability to anxiety. Scores on the trait subscale are elevated in individuals who meet criteria for anxiety disorders (AD), across subtypes (Bieling et al., 1998; Chambers et al., 2004). Elevated trait anxiety scores on the STAI questionnaire have also been shown to predict future AD diagnosis (Plehn and Peterson, 2002). Participants completed the STAI trait anxiety subscale at the beginning of the experimental session prior to data collection. In the sample as a whole, STAI trait anxiety scores ranged from 20 to 57 (*M* = 39.2, *SD* = 8.46). In the sub-sample who completed the fMRI task, STAI trait anxiety scores ranged from 22 to 57 (*M* = 39.5, *SD* = 9.75). These scores are comparable to published norms for this age group (Spielberger et al., 1983).

The behavioral task was conducted in a quiet testing room, the fMRI task in UC Berkeley's 3T MRI facility. Participants who participated in the combined fMRI and behavioral session completed the fMRI task prior to the behavioral task with a short break in between. Behavioral task only participants completed the behavioral task immediately following administration of the STAI.

### Experimental stimuli

**Figure 1:**
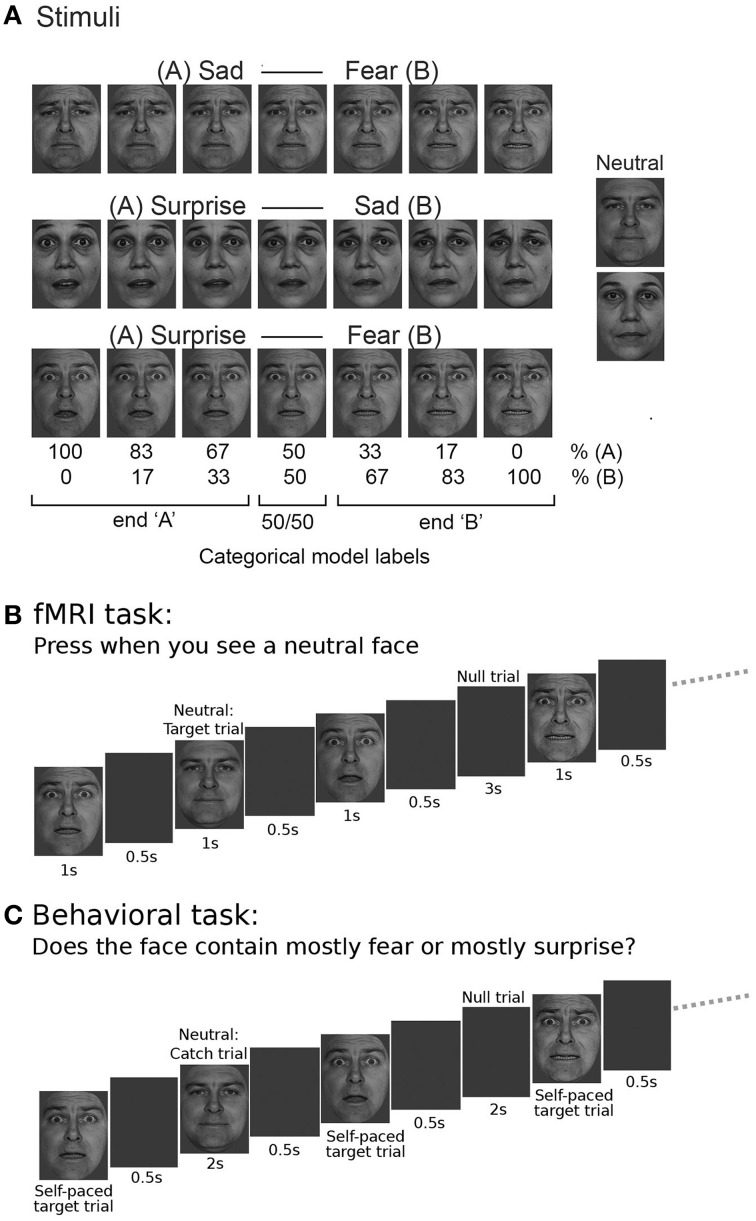
**Task stimuli and design. (A)** Three emotional expression continua were created by morphing between sad and fearful, surprised and sad, and surprised and fearful expressions, using six 16.7% morph steps. These continua were created for both a male and a female actor, faces were taken from the Pictures of Facial Affect set (Ekman and Friesen, 1976). The actors' neutral expressions were also used as stimuli, but not included in the continuum construction. For the main fMRI analysis, using a categorical model, faces were labeled according to the end of the continuum to which they belonged (“A” or “B,” e.g., “surprise” or “fear”) with a third label being given to “50/50” morphs that comprised equal amounts of each end expression. **(B)** Within the fMRI task, participants were shown faces with expressions from one continuum at a time, presented together with neutral faces and double length null trials in a pseudo-random fashion using a “type 1 index 1 sequence” (Figure S1). Participants were asked to respond by key press to presentation of the neutral face. **(C)** In the behavioral task, an equivalent type 1 index 1 presentation sequence was used but participants were asked to respond to all faces except the neutral trials, categorizing the faces as showing “mainly” expression “A” or expression “B” for a given continuum (e.g., “mainly surprise” or “mainly fear” for the surprise-fear continuum, as shown in the example.)

Faces were taken from the Pictures of Facial Affect (POFA) set (Ekman and Friesen, 1976). Faces showing fearful, sad, and surprised expressions were selected for two different actors (one male, one female). These were used to construct emotional expression morphs for each identity. Each pair of emotional expressions was morphed separately using the Fantamorph software package (Abrosoft Inc.) This yielded a continuum of morphed faces for each original pair of expressions: surprise-fear, sad-fear, and surprise-sad. Each continuum comprised pure expressions at the end of each continuum (e.g., 100% fear, 0% surprise, and 0% fear, 100% surprise) and five intermediate blended expressions that differed from each other in 16.7% morph steps (e.g., 16.7% surprise, 83.3% fear; 33.3% surprise, 66.7% fear … 83.3% surprise, 16.7% fear), Figure 1A. The neutral expressions of the two actors were used as target stimuli in the fMRI task (and catch trials in the behavioral task) in order to maintain vigilance (see below) but were not involved in the construction of any of the morph continua.

### fMRI task

Stimuli were presented in an unbroken, counterbalanced sequence. This allows adaptation effects to be investigated on every trial as a function of the difference between the current and the previous stimulus (Aguirre, 2007). In each imaging run, participants were presented with stimuli from a single expression continuum (e.g., the surprise-fear continuum). Presentation of faces from a single continuum at a time enabled us to examine adaptation effects as a function of transitions along a given continuum. For each of the two identities, each of the seven levels of expression (100% expression A, 0% expression B, 83% expression A, 17% expression B …, 0% expression A, 100% expression B) together with neutral face trials and “null” trials (blank screen only) were presented using a “type 1 index 1” sequence of 82 trials (Finney and Outhwaite, 1956; Aguirre, 2007), Figure S1. This refers to a sequence of length *n*^2^ +1 trials where there are *n* conditions or trial types and there is first order counterbalancing, specifically each condition follows every other condition and itself once. In addition, each of the *n* trial types occurs once in pseudo-random order within each sequential set of *n* trials minimizing differential habituation across trial types (the only detectable regularity being that each new set of *n* trials starts with the same trial type that the previous set finished with).

The sequence for one identity was concatenated with the sequence for the other identity with a 12 s gap in between. The second sequence was pseudo-randomized independently and was not identical in order to the first. We only used two identities and did not mix identities within each sequence to avoid confounds resulting from fluctuations in strength of expression between identities. Including a mixture of identities would also have entailed many more trials to separate effects of identity-related adaptation from expression-related adaptation. Each face was presented for 1 s followed by a blank screen for 500 ms (Figure 1B). Null trials lasted for 3 s (i.e., twice the duration of face trials, enhancing power for detection of main effects; Aguirre, 2007). Participants were asked to press a button with the right index finger any time the neutral expression occurred (i.e., no responses were made on the trials of interest), and to pay attention to all the facial expressions shown. The occurrence of neutral face trials was not predictable, requiring participants to maintain concentration. Before the start of each imaging run, participants were shown the target neutral faces for both identities. There were nine runs, three per expression continuum. Prior to the start of the fMRI session, participants performed short practice blocks for each expression continuum outside the scanner. This ensured that participants were familiar with the facial emotional expressions presented during the fMRI session and with the task itself.

### Behavioral task

Vision Egg (Straw, 2008) was used for stimulus delivery, as for the fMRI task. The task was performed on a desktop computer in a behavioral testing room, with the visual angle subtended by the face stimuli equated to that within the fMRI task (5 × 8 degrees). The stimuli used were the same as in the fMRI task, and were also presented using type 1 index 1 sequences of 82 trials. However, the task performed was different. Participants were instructed to judge the main emotion contained in the facial expression shown on a given trial, while avoiding responding to neutral faces (i.e., here neutral face trials acted as catch trials), Figure 1C. For example, in the surprise-fear continuum, participants were asked to judge if the presented face showed “mainly surprise” or “mainly fear.” Responses were made by key press. The emotions chosen between were mapped onto unique keys on the keyboard (color labeled for easier task performance). Mappings were randomized across participants. At the beginning of each task block, participants were told which two emotions they would be categorizing the faces according to and reminded of the color of the corresponding key for each emotion. Participants performed training blocks for each continuum to familiarize them with the task and emotion key mapping. Trials were self-paced with participants having a maximum of 6 s to make a response (mean reaction time = 760 ms). Neutral face catch trials were shown for 2 s. Null trials lasted 2 s. A blank period of 4 s occurred in between the two facial identities shown in each task block. Participants completed six task blocks, two per expression continuum. Each task block contained two sets (one per identity) of 82 trials of faces corresponding to nine repetitions of each of the different “morph levels” from a given continuum (plus neutral and null trials). These were presented using type 1 index 1 sequences starting with a double null trial as in the fMRI task.

### fMRI data acquisition

Blood oxygenation level dependent (BOLD) contrast functional images were acquired with echo-planar T2^*^-weighted imaging (EPI) using a Siemens Tim Trio 3T MR system with a 12 channel head coil. Each image volume consisted of 26 sequential 3 mm thick slices (interslice gap, 0.75 mm; in-plane resolution, 2.4 × 2.4 mm; field of view, 234 by 234 mm; matrix size, 98 by 98; flip angle, 74 degrees; echo time, 33 ms; repetition time, 2 s). Slice acquisition was transverse oblique, angled to avoid the eyes, and provided near whole brain coverage. These acquisition parameters were chosen to minimize voxel size while covering all brain regions of interest. In some subjects, cerebellum and part of motor cortex was not covered by our slice prescription. To aid co-registration, an additional eight echo-planar volumes were acquired using the same parameters as the task data but with an increased number of slices and adjusted TR. Data were acquired in nine scanning runs lasting approximately 5 min each. The first five volumes of each run were discarded to allow for T1 equilibration effects. A T1-weighted structural scan was also acquired at 1 mm isotropic resolution. This was acquired prior to the functional data.

### fMRI preprocessing

Data were preprocessed using Matlab version 7.3 (The MathWorks, Natick, MA) and SPM5 (Welcome Department of Imaging Neuroscience, London, UK). After conversion from DICOM to NIfTI format, diagnostics were run on the time series for each imaging run. Following an approach similar to that adopted by Power et al. (2012) and Carp (2013), bad volumes (with unusually high changes in mean whole brain signal intensity) were replaced by the average of the volumes on either side. These volumes were identified using the SPM timeseries diagnostic tool tsdiffana.m. Among other indices, this calculates the mean square difference of voxel-wise signal intensities, averaged across the whole volume, between each volume (*n*) and the previous volume (*n* − 1) and divides this by the mean signal across the whole volume averaged over the whole timeseries. Volumes (both *n* and *n* − 1) were rejected using an absolute cutoff (the recommended default of 10) as this handled differences between participants in the noisiness of data better than a within-participant percentile cut off. In line with findings by Power et al. (2012), bad volumes tended to correspond to those with notable spikes in movement. For each pair of volumes replaced, a “bad scan” regressor of no interest that coded these volumes as 1 and all other volumes as 0 was created to model out the replaced volumes in the final analysis.

Subsequent to this initial data-cleaning step, image realignment (correcting for head movement) was conducted, followed by slice time correction. The subject's T1-weighted structural scan was aligned to their EPI data. Following this, the T1 was transformed into standard (MNI) space using SPM5's combined segmentation and normalization procedure (Ashburner and Friston, 2005) and the transformation applied to the echo-planar images. These images were resampled to 2 mm isotropic voxels. A high-pass filter of 180 s was used to remove low-frequency scanner noise. Spatially unsmoothed images were used for region of interest (ROI) based analyses, see below. Whole-brain voxelwise analyses were used to define functional ROIs implicated in face processing (also see below). For these analyses, spatial smoothing of the echoplanar images was conducted with a Gaussian kernel with a full-width-to-half-maximum value of 6 mm.

### fMRI data analysis

**Figure 2:**
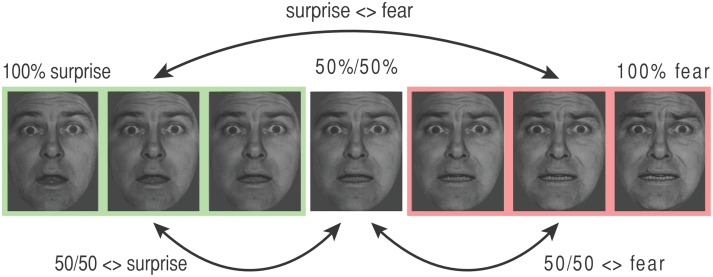
**Key transitions coded by adaptation regressors in the categorical model**. The stimulus presented on each trial “*n*” was labeled using both direct effect and adaptation regressors. Direct effect regressors code stimulus type. For the categorical model, all faces with 67%+ expression “A” were coded as “end A” (e.g., “surprise,” shown in green), those with 67%+ expression “B” were coded as “end B” (e.g., “fear,” shown in pink), and the 50/50 morph was coded using a third separate regressor. Adaptation regressors describe the difference between the stimulus on trial *n* and the stimulus on trial *n* − 1. These are illustrated here for the surprise-fear continuum. “Surprise <> fear”: transitions between any face from the surprise (green, “A”) end of the continuum and any face from the fear (pink, “B”) end of the continuum, where either face type can occur at trial *n* − 1 or trial *n* (note, all adaptation effects are coded symmetrically). (ii) “50/50 <> fear”: transitions between a 50/50 surprise/fear morph and a fear (“B”) end face, (iii) “50/50 <> surprise”: transitions between a 50/50 surprise/fear morph and a surprise (“A”) end face. Adaptation regressor values for the additional graded (linear) adaptation model can be found in Figure S2.

The data from each expression continuum were modeled separately. Events were modeled by a delta (impulse) function. The regressors of interest used can be divided into those representing direct stimulus effects (i.e., the “condition” to which the stimulus on a given trial (*n*) belonged, as commonly used in most experiments) and those representing “carry over” or adaptation effects. The latter were coded in terms of the difference or distance between the stimulus on the current trial (*n*), and the stimulus on the prior trial (*n* − 1), though the exact nature of this varied between the two models used (see below and Figure 2; Figure S2). A number of regressors of “non-interest” were also included in both models to remove task-unrelated variance (noise). These comprised six realignment (movement) regressors, regressors indicating volumes where “bad scans” had been replaced by the average of neighboring volumes, and mean time-series extracted from outside-of-brain masks.

Regressors of interest were convolved with the canonical HRF function and together with the (noise) regressors of non-interest were used to model the mean BOLD response from right and left amygdala ROIs (defined using the Montreal Neurological Institute Automated Anatomical Labeling template, Tzourio-Mazoyer et al., 2002). Non-smoothed data were used; analysis was conducted with the MARSBAR ROI toolbox for SPM (Brett et al., 2002). Additional ROI analyses are reported for functionally defined ROIs from across the face-processing network (see below). While our a-priori hypotheses focused on the amygdala, the results of these additional analyses are provided for researchers with more general interests in the differentiation of aspects of face processing across different brain regions.

#### Model overview: categorical vs. graded

There has been much debate as to whether the perception of facial expressions is categorical or continuous in nature. Behaviorally, the categorization of faces from expression morph continua of the type used in the current study has been found to fit well with a categorical model, typically showing a sigmoidal function with a sharp boundary transition between judgments of morphed faces as showing one emotion or another (Etcoff and Magee, 1992; Calder et al., 1996; Young et al., 1997; Said et al., 2010). However, expressions further from prototypes (i.e., 100% expressions) take longer to categorize and participants can discriminate between exemplars categorized as showing the same expression (Young et al., 1997). Within the fMRI literature, it has been argued that the amygdala shows categorical representation of facial expression but that other regions in the face processing network may show more graded or continuous representation of expressions or associated changes in physical features (Said et al., 2010; Harris et al., 2012). Given the dominance of the categorical model in the behavioral literature combined with our primary focus on the amygdala, we adopted a categorical model for our main fMRI analyses. However, we also constructed a continuous, graded, model to enable additional examination of linear adaptation effects as a function of “morph steps” along the expression continua, this is described following the main categorical model.

#### The categorical model

Data from each expression continuum were modeled independently. According to a categorical model there should be a sharp transition in representation of faces from expression “A” to expression “B” with little difference in representation within expressions labeled as “A” or “B.” In order to model a categorical or step function, stimuli were dichotomously allocated to the end of the expression continuum to which they were closest. So, for example, for the surprise-fear continuum, faces with 100% surprise, 0% fear; 83% surprise, 17% fear; and 67% surprise, 33% fear were allocated to the “surprise end,” and faces with 33% surprise, 67% fear; 17% surprise, 83% fear; and 0% surprise, 100% fear) were allocated to the “fear end” (Figure 1A, also see Figure 2). 50/50 morphs were modeled using a third direct effect regressor. The regressors of primary interest, however, were the adaptation regressors. For each trial “*n*” these encoded the nature of the transition from the stimulus shown on the previous trial (*n* − 1) to that shown on the current trial (*n*). There were three primary adaptation regressors (Figure 2). The first represented transitions between one end of a given continuum and the other. In the surprise-fear continuum, for example, if trial “*n*” was a face with 66% or greater surprise (“surprise end”) and trial *n* − 1 was a face with 66% or more fear (“fear end”), or vice versa, this “surprise <> fear” regressor was given a “1” on trial *n*, otherwise it was given a “0.” The second adaptation regressor coded transitions between 50/50 morphs and faces from the right-hand end “B” of a given continuum. This second regressor (e.g., “50/50 <> fear”) was also directionless, i.e., trial “*n*” was given a “1” for this regressor regardless of whether it was a fear end face following a 50% surprise/50% fear face or a 50% surprise/50% fear face following a fear end face. The third adaptation regressor coded for transitions between 50/50 morphs and faces from the left-hand end “A” of a given continuum (e.g., “50/50 <> surprise” in the case of the surprise-fear continuum).

We were interested in testing the proposal that elevated trait anxiety would be associated with a bias toward representing 50/50 morphs containing fear more similarly to expressions from the fear end of the surprise-fear and sad-fear continua than to expressions from the non-fear ends of these continua. If this is the case, then individuals with high trait anxiety scores should show more adaptation for 50/50 <> fear end face transitions than for 50/50 <> sad or surprise end face transitions for these two continua.

For completeness, two additional direct effect regressors were included—one modeled the occurrence of neutral face (target) trials and one the occurrence of any emotional expression, regardless of type. Four additional adaptation regressors were also included—these modeled direct repetitions of a given expression blend, direct repetition of the neutral face, trials where any emotional face followed a neutral face and trials where any emotional face followed a null trial.

### Graded non-categorical model

Supplementary use of a graded non-categorical model allowed us to examine whether particular brain regions showed continual graded representation of the expressions that made up each morph continuum—i.e., whether they linearly tracked changes in the stimuli, as measured in morph steps. Such a model is expected to fit well in regions sensitive to low-level physical changes in face stimuli, regardless of whether these changes contribute to changes in percept. The Occipital Face area (OFA)—a right-lateralized region of lateral occipital cortex (Gauthier et al., 2000)—is thought to be such a region.

The graded model contained a direct effect regressor for “morph level” (0–100% in 16.7% increments or steps along the continuum of interest). A second direct effect regressor modeled the occurrence of neutral faces and a third the occurrence of any face with an emotional expression. The key adaptation regressor coded the distance of the stimulus presented on trial *n* in morph steps (in either direction) from the stimulus presented on the previous trial (*n* − 1), Figure S2. Two additional adaptation regressors modeled direct repeats of a given expression blend and direct repeats of neutral faces, two others indicated when faces with any emotional expression followed a neutral face (emotion after neutral) or a null trial (emotion following null).

### Functional definition of additional ROIs implicated in face processing

Regions of interest for the Occipital Face Area (OFA), Fusiform Face Area (FFA, Kanwisher et al., 1997), and Superior Temporal Sulcus (STS) were functionally defined using the graded non-categorical model and contrasts orthogonal to those in which we were interested. Activation to presentation of any emotional expression, including those following null trials, but not those following neutral (target) trials, was used to define bilateral FFA and STS ROIs. Group-level maps thresholded using family-wise error (FWE) whole brain correction for multiple comparisons at *p* < 0.05 were used to identify activation clusters of interest. To have a similar number of voxels in each ROI, we used slightly different thresholds for right FFA and right STS (*t* > 7 and *t* > 5.25, respectively). The right FFA cluster comprised 213 voxels with activation peak: *x*, *y*, *z*: 40, −48, −18 (MNI space). The right STS cluster comprised 221 voxels and had its peak at *x*, *y*, *z* = 56, −38, 12. This contrast also yielded an activation cluster in right lateral occipital cortex, although this was too diffuse to allow identification of a constrained region corresponding to OFA as described in previous reports. Activation to neutral target faces vs. baseline (also an orthogonal contrast to those of interest) did however allow for definition of a constrained right OFA ROI that corresponded well to previous accounts (Pitcher et al., 2011a). The group level activation map for this contrast was thresholded at *t* > 13, giving a cluster of 224 voxels, with activation peak: *x*, *y*, *z* = 40, −86, −4. No clear left lateralized activation was observed, in line with prior findings indicating greater involvement of right hemisphere regions in face processing, hence these right ROIs were mirrored across the sagittal plane to create corresponding left lateralized ROIs.

## Results

### Behavioral results

As described above, subjects performed a two-way classification of emotional expressions from three morph continua (surprise-fear, sad-fear, and surprise-sad). In each continuum, the faces to be classified were morphs between exemplars of the end expressions (two distinct identities were used, with seven morph levels including the two pure end expressions, Figure 1A). Given behavioral effects are often smaller and noisier than fMRI effects, we combined data from participants who performed both the fMRI and behavioral task with that from participants who performed the behavioral task alone. This gave us a combined sample size of 54. Generalized mixed effects logistic regression analyses were conducted using the R lme4 package, R Development Core Team (2011), separately, for categorization data from each expression continuum. Covariates of interest comprised participant trait anxiety (continuous), morph “level” (0–100% expression “B” in 16.7% increments) and the interaction term for morph level × trait anxiety. Participant ID (nominal) was additionally entered as a random effects term. Behavioral categorization of the faces from each continuum (as expression “A” vs. expression “B”) provided the binary dependent variable.

**Figure 3:**
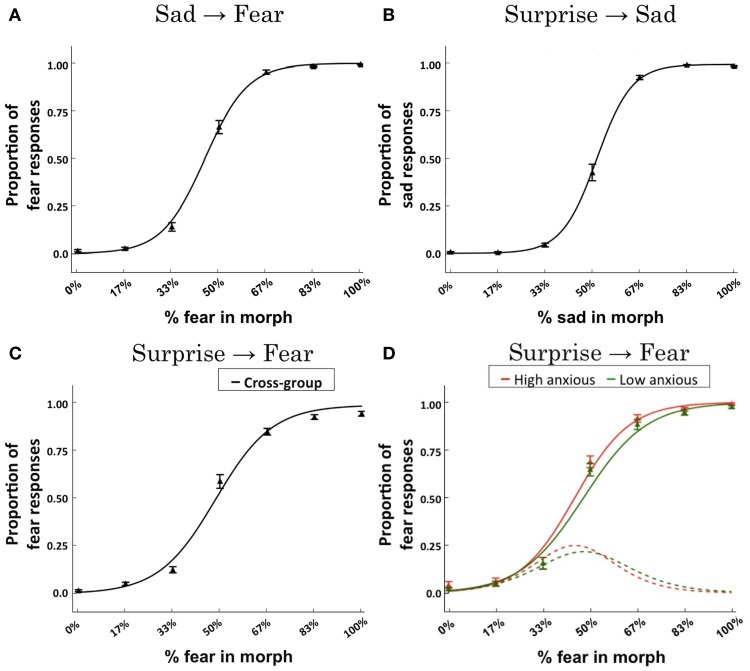
**Behavioral results**. Logistic regression fits across subjects for each of the expression continua: **(A)** sad-fear; **(B)** surprise-sad; **(C)** surprise-fear. Data points indicate the mean proportion of responses across subjects (±SEM) made for each expression morph with reference to the right-ward (“B”) end of the continuum—i.e., proportion of fear responses for the sad-fear and surprise-fear continua, proportion of sad responses for the surprise-sad continuum. The solid line represents the logistic regression fit to the group data. **(D)** A mixed effects logistic regression model revealed an interaction of trait anxiety x morph content (% fear in morph) for the surprise-fear continuum, β = 0.056, *p* = 0.0248. To illustrate the effect of anxiety upon categorization responses as a function of morph level, the logistic regression fit is presented for trait anxiety levels ±two standard deviations from the mean (this corresponds to scores of 20 and 59, respectively, the mean STAI trait score being 39.5, *SD* = 9.75). These fits are plotted using solid lines. Red = high anxious. Green = low anxious. The derivatives of these fitted functions (which gives the rate of change in responses from surprise to fear) are presented using dotted lines. Data points (triangles) for individuals with trait anxiety scores in the top tertile (red) and bottom tertile (green) of the group are also shown. It can be seen that heightened anxiety is associated with a more rapid transition from surprise to fear responses with this difference emerging for morphs with 33–50% fear content (see the derivative plots) and leading to more fear responses being made by high anxious individuals until high levels of fear content (83–100%) are reached, at which point both high and low trait anxious individuals predominantly make fear judgments (see the main fits).

These regression analyses revealed a significant effect of “morph level” upon categorization responses within each continuum (*p*s < 10^−11^). For each continuum, the categorization function showed the expected sigmoidal response form (Figures 3A–C). Across subjects, the slope of the categorization function for the surprise-fear continuum was less steep than that for either the surprise-sad or sad-fear continua, *t*_(53)_ = 2.48, *p* = 0.017, *t*_(53)_ = 2.96, *p* = 0.005, respectively. The slopes of the categorization functions for surprise-sad and sad-fear did not differ significantly from each other, *t*_(53)_ = 0.71, *p* = 0.481.

In these behavioral analyses, our question of primary interest was whether trait anxiety would modulate the transition from non-fear to fear judgments in the surprise-fear and sad-fear continua, as a function of increasing percentage of fear in the expression shown. This was observed to be the case in the surprise-fear continuum alone, interaction of trait anxiety × morph content (% fear in morph), β = 0.056, *p* = 0.0248. As can be seen in Figure 3D, individuals with elevated trait anxiety showed an earlier, sharper, transition from categorizing expressions as surprise to categorizing them as fear, as a function of the percentage of fear in the face presented, leading to increased fear categorizations for faces in the middle of the surprise-fear continuum. There was no significant effect of trait anxiety upon categorization performance as a function of morph level within the sad-fear or surprise-sad continua.

### fMRI results

**Figure 4:**
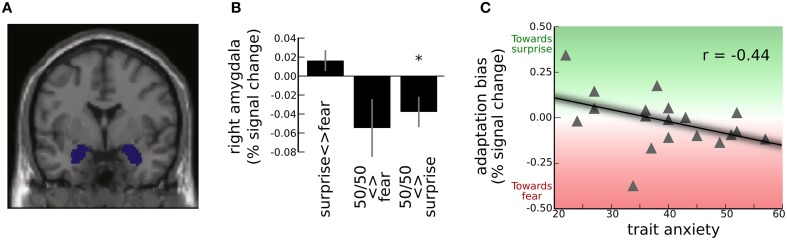
**Adaptation effects in the amygdala for the surprise-fear continuum. (A)** Activation was extracted and averaged from left and right amygdala ROIs, shown here. These ROIs were specified using the MNI Automated Anatomical Labeling Template (Tzourio-Mazoyer et al., 2002). **(B)** Group level amygdala adaptation effects (mean and S.E.) for trial-to-trial transitions, as revealed by the categorical model. Significant adaptation of the right amygdala response (decrease in BOLD signal) was observed for transitions between 50/50 surprise/fear morphs and faces from the surprise end of the surprise-fear continuum (“50/50 <> surprise,” see Figure 2) *Group-level one sample t-test against zero, *t*_(18)_ = −2.37, *p* <0.05. For transitions between 50/50 surprise/fear morphs and faces from the fear end of the surprise-fear continuum (“50/50 <> fear”), there was higher inter-individual variability and the group level adaptation effect did not reach significance. There was slight but non-significant release from adaptation for transitions between faces from one end of the continuum and the other (“surprise <> fear”). **(C)** “Adaptation bias” refers to the extent to which adaptation was greater for transitions between 50/50 morphs and fear end faces than between 50/50 morphs and surprise end faces (i.e., “50/50 <> fear”-“50/50 <> surprise”). This was significantly modulated by anxiety, *r*_(17)_ = −0.44, *p* < 0.05. High trait anxious individuals showed greater adaptation (i.e., decrease) of the right amygdala BOLD response when 50/50 surprise/fear faces followed or preceded faces from the fear (vs. surprise) end of the continuum, this effect reversing in low trait anxious individuals.

In line with previous studies, our behavioral data are consistent with a categorical model of representation of emotional expressions. While this may not translate to categorical representation across the face-processing network, given our a-priori focus on the amygdala and our desire to understand biases in behavioral categorization performance, we focused primarily on the categorical model to test our hypotheses regarding the modulation by trait anxiety of representation of expression blends—especially those containing fear—within the amygdala. We used a region of interest approach, activity being extracted from, and averaged across, left and right amygdala ROIs defined using the MNI AAL template (Figure 4A). This approach avoids issues pertaining to corrections for multiple comparisons and inflated effect sizes when reporting peak voxel statistics following whole-brain or small-volume search (Vul et al., 2009). One-tailed tests at *p* < 0.05 were used to test a-priori hypotheses. Other results are reported as significant if they survived *p* < 0.05, two-tailed.

#### Adaptation effects in the amygdala

We first examined the extent to which amygdala activity showed release from adaptation when emotional faces followed a neutral face. If the amygdala shows preferential activation to emotional faces, in general, relative to neutral faces then we would expect to see such a rebound in activity. This was indeed observed across continua, left amygdala: *t*_(18)_ = 3.63, *p* = 0.002, right amygdala: *t*_(18)_ = 2.38, *p* = 0.029. For both amygdala ROIs, this effect did not vary significantly by continuum (*p*s > 0.4), nor was it modulated significantly by anxiety across or within continua (*p*s > 0.5).

#### Amygdala adaptation for transitions involving 50/50 morphs: group level effects

Using the categorical model, the relative magnitude of amygdala adaptation for transitions between 50/50 morphs and faces from one end, vs. the other, of a continuum, can give us a metric of whether the representation of 50/50 morphs is “biased” toward one end of the expression continuum. For the surprise-fear continuum, participants as a group showed significant adaptation of the amygdala response for transitions between 50/50 morphs and faces from the surprise end of the continuum; “50/50 <> surprise”: left amygdala, *t*_(18)_ = −3.86, *p* = 0.001, right amygdala, *t*_(18)_ = −2.37, *p* = 0.029, Figure 4B. At this group level, there was also a non-significant trend toward adaptation of the amygdala BOLD response for transitions between 50/50 morphs and faces from the fear end of the continuum in the right amygdala, “50/50 <> fear”: *t*_(18)_ = −1.80, *p* = 0.08. The left amygdala showed no significant adaptation for this transition type, *p* > 0.1. The difference in BOLD adaptation for “50/50 <> fear” transitions vs. “50/50 <> surprise” transitions did not reach significance in either left or right amygdala, *p*s > 0.1. In other words, there was no strong evidence at the group level for a bias in representation in the amygdala response to 50/50 morphs toward either the “surprise” or the “fear” end of the continuum. It is also of note that there was considerable individual variability around the group mean for “50/50 <> fear” transitions, Figure 4B.

For the sad-fear continuum, participants as a group showed significant adaptation of the left amygdala response for transitions between 50/50 morphs and faces from the sad end of the continuum, “50/50 <> sad”: left amygdala, *t*_(18)_ = −2.25, *p* = 0.038, this was not significant in the right amygdala, *p* > 0.1. At the group level, no significant amygdala adaptation was observed for transitions between 50/50 morphs and faces from the fear end of the continuum, “50/50 <> fear”: *p*s > 0.1, and there was a non-significant trend toward greater adaptation for 50/50 <> sad transitions than for 50/50 <> fear transitions in the left amygdala, *t*_(18)_ = 1.87, *p* = 0.078.

For the surprise-sad continuum, participants as a group showed a non-significant trend toward adaptation of the right amygdala response for transitions between 50/50 morphs and faces from the sad end of the continuum, “50/50 <> sad”: *t*_(18)_ = −1.89, *p* = 0.075, and a trend toward greater adaptation for “50/50 <> sad” transitions than for “50/50 <> surprise” transitions in the left amygdala, *t*_(18)_ = −1.86, *p* = 0.079. No other effects reached or trended toward significant, *p*s > 0.1.

#### Anxiety-related bias in representation of 50/50 expressions as indexed by amygdala BOLD adaptation

We come now to the primary analyses of interest. The two main hypotheses we tested were that (1) elevated trait anxiety would be associated with greater adaptation of the amygdala BOLD response for transitions between 50/50 morphs and expressions from the “fear” end of the surprise-fear and sad-fear continua than for transitions between these 50/50 morphs and expressions from the non-fear ends of these continua, and (2) this would either be observed for (i) both of these continua or (ii) only the surprise-fear continuum where effects of trait anxiety were observed upon categorization behavior.

Our first hypothesis predicted that we should observe a directional difference in adaptation or “adaptation bias” as a function of trait anxiety. For the surprise-fear continuum, “adaptation bias” was calculated as BOLD adaptation for “50/50 <> fear” transitions minus BOLD adaptation for “50/50 <> surprise” transitions (Figure 2). This adaptation bias score varied significantly with participant anxiety, *r*_(17)_ = −0.44, *p* = 0.031, one-tailed, Figure 4C. Here, the sign for the correlation coefficient is negative, reflecting a greater decrease in BOLD signal—more adaptation—for “50/50 <> fear” transitions than for “50/50 <> surprise” transitions as a positive function of trait anxiety. In other words, in line with our predictions, high trait anxious individuals showed a pattern of activity consistent with greater representational similarity between 50/50 surprise/fear expressions and expressions from the fear end of the continuum, while low trait anxious individuals showed a pattern of activity consistent with greater representational similarity between 50/50 surprise/fear expressions and expressions from the surprise end of the continuum.

In contrast to the findings for the surprise-fear continuum, no association between trait anxiety and “adaptation bias” was observed in the sad-fear continuum, *p*s > 0.1 (here this was calculated as amygdala adaptation for 50/50 <> fear end face transitions minus amygdala adaptation for 50/50 <> sad end face transitions). This parallels the behavioral findings where anxiety-related differences in categorization behavior were only observed in the surprise-fear and not the sad-fear continuum, and as such supports hypothesis 2ii.

The third a-priori question we addressed, was whether an effect of trait anxiety would be seen upon amygdala adaptation for faces from the surprise-sad continuum, where no percentage of “fear” was present in any expression. There was no significant relationship between trait anxiety and magnitude of amygdala adaptation bias in this continuum, *p*s > 0.4. It should also be noted, that for all three continua, there was no effect of trait anxiety upon the extent of adaptation observed when faces with expressions from one end of a given continuum followed faces from the other end of the same continuum, *p*s > 0.2.

#### Relationship between neural and perceptual representational bias

Only a subset of participants who completed the behavioral session also completed the fMRI session. This limits our power for examining brain—behavior relationships. Nevertheless, there was evidence of a non-parametric correlation between “adaptation bias” in the right amygdala and the slope of the surprise-fear categorization function fitted to participants' categorization of faces from the surprise—fear continuum as showing either surprise (0) or fear (1), Spearman's *r*_(17)_ = −0.45, *p* = 0.027, one-tailed. This relationship reflects an association, across participants, between the sharpness of transition from categorizing faces as surprise to as fear (as a function of the % of fear in the face) and the extent to which greater amygdala adaptation (negatively signed to reflect a decrease in BOLD activity) was observed for 50/50 <> fear face transitions than for 50/50 <> surprise face transitions. It is of note that elevated anxiety was associated with both a sharper transition in the behavioral categorization of faces from the surprise-fear continuum and increased amygdala “adaptation bias.” Within the surprise-sad continuum, there was also a non-parametric correlation between right amygdala adaptation bias and the slope of the behavioral categorization function, *r*_(17)_ = −0.58, *p* = 0.005, one-tailed, but neither of these indices were correlated with trait anxiety, *p*s > 0.1. There was no significant relationship between amygdala adaptation bias and slope of the behavioral categorization function for the sad-fear continuum, *p*s > 0.1. There was also no significant relationship between adaptation bias in FFA and the slope of the behavioral categorization function for any of the three expression continua, *p*s > 0.1.

#### No evidence for linear adaptation to expression within the amygdala

In line with our expectation that the amygdala response would best be characterized by a categorical model, application of the graded model (see Materials and Methods, Figure S2) revealed no significant evidence for linear adaptation effects as a function of change in morph expression level (0–6 “steps”) between the prior and current trial, for any of the three continua within either left or right amygdala (*p*s > 0.1). In addition trait anxiety did not interact significantly with this linear adaptation term (*p*s > 0.1).

#### Findings from the extended face network

##### Transition from graded to categorical representation of emotional expressions along the ventral visual stream

**Figure 5:**
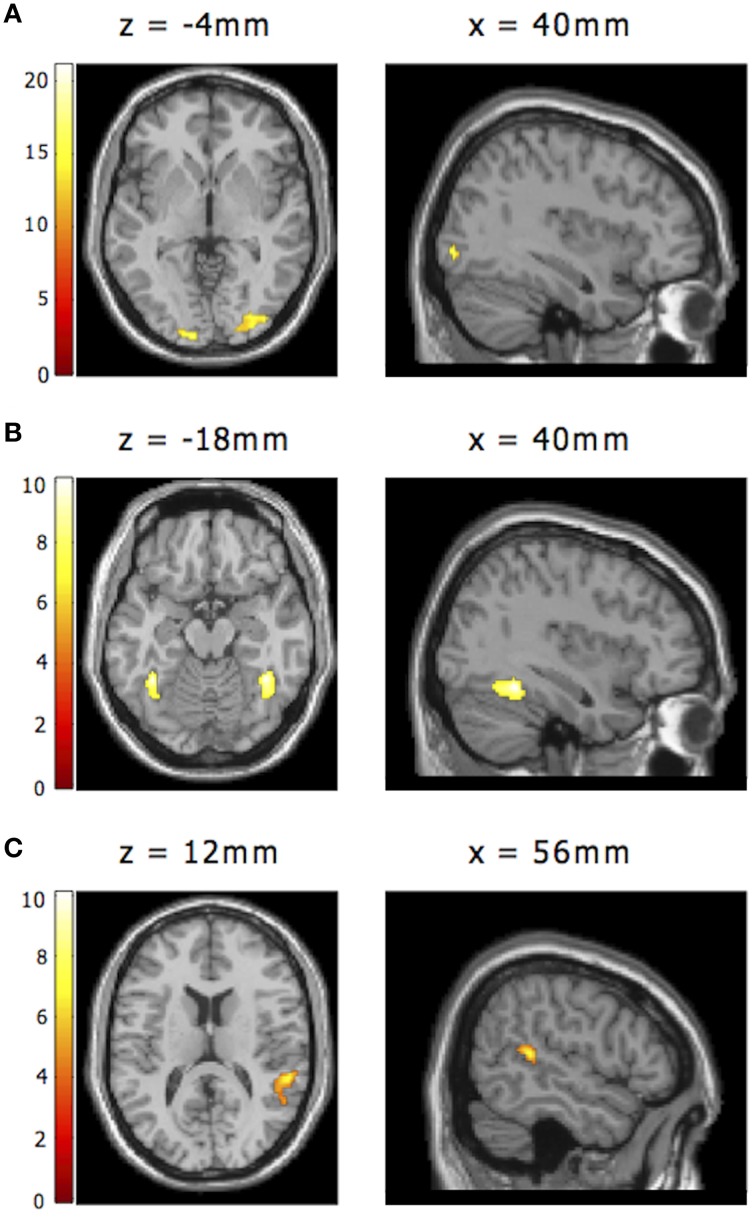
**Additional regions of interest from the face processing network. (A)** Right OFA as functionally defined by activity to neutral faces vs. baseline, using the graded model (statistical map thresholded at *t* > 13; activation peak: *x*, *y*, *z* = 40, −86, −4). **(B)** Right FFA as functionally defined by activity to any emotional face including emotional faces after nulls (statistical map thresholded at *t* > 7; activation peak: *x*, *y*, *z*: 40, −48, −18). **(C)** Right STS as functionally defined by activity to any emotional face including emotional faces after nulls (statistical map thresholded at *t* > 5.25; activation peak: *x*, *y*, *z*: 56, −38, 12). *Note*. These contrasts were orthogonal to our analyses of interest, as reported within the Results section. Right lateralized ROIs for OFA, FFA, and STS were mirrored across the sagittal plane to create corresponding left lateralized ROIs (not shown).

We used data from across the extended face network (see Materials and Methods, Figure 5) to address a number of additional questions. First, we were interested in the extent to which adaptation effects in OFA, FFA, and STS would be consistent with representation of facial expression becoming increasingly categorical as we moved up the ventral visual processing stream. Both linear changes in release from adaptation as a function of physical change in stimuli between trials (graded model) and, especially, sensitivity (as indexed by release from adaptation) to changes within each end of the expression continua (categorical model) would be consistent with processing of the physical changes in facial features that characterize each step between morph levels as opposed to altered representation of the actor's emotional state.

**Figure 6:**
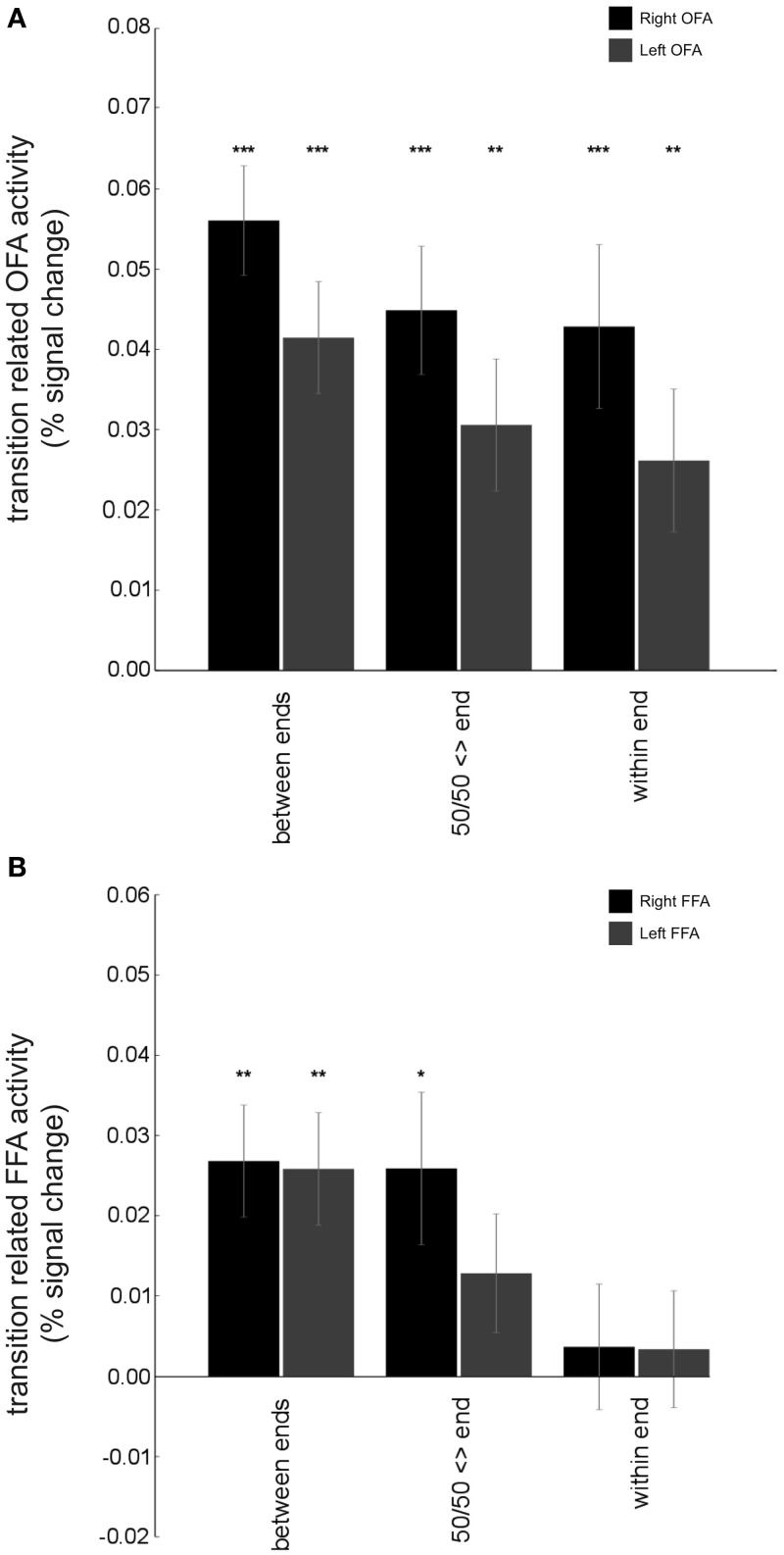
**Contrasting adaptation effects in OFA and FFA: results from the categorical model. (A)** The BOLD response for both right and left OFA (averaged across each ROI) showed significant release from adaptation not only for transitions from one end of a continuum to the other (“between end” transitions), right OFA: *t*_(18)_ = 8.21, *p* < 0.0001, left OFA: *t*_(18)_ = 5.94, *p* < 0.0001, but also for transitions between 50/50 morphs and expressions from either end of the continua (“50/50 <> end” transitions), right OFA: *t*_(18)_ = 5.61, *p* < 0.0001, left OFA: *t*_(18)_ = 3.72, *p* < 0.005, and for transitions between expressions within a given end of a continuum (“within end” transitions), right OFA: *t*_(18)_ = 4.21, *p* < 0.001, left OFA: *t*_(18)_ = 2.94, *p* < 0.01. This is consistent with the OFA representing physical differences in expressions even when they are perceived similarly. **(B)** Both right and left FFA showed significant release from adaptation of the BOLD response for “between end” transitions, right FFA: *t*_(18)_ = 3.85, *p* < 0.005, left FFA: *t*_(18)_ = 3.69, *p* = 0.005. For “50/50 <> end” transitions, release from adaptation only reached significance in right FFA, right FFA: *t*_(18)_ = 2.73, *p* < 0.05, left FFA: *t*_(18)_ = 1.73, *p* = 0.10. Further, no significant release from adaptation was observed for “within end” transitions, *p*s > 0.5. These findings suggest that representation of expressions in OFA may be more graded and less categorical in nature than that within FFA. Bars show group mean (±S.E.) One sample *t*-tests against 0, ^*^p < 0.05, ^**^p < 0.01, ^***^p < 0.001.

**Figure 7:**
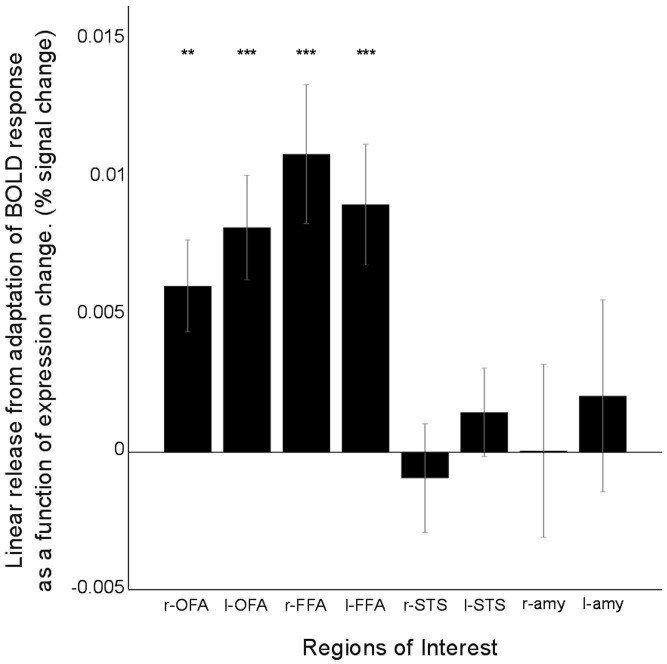
**Differences in linear adaptation effects across regions: results from the graded model**. Bilateral OFA and FFA showed significant linear release from adaptation of the BOLD response as a function of percentage change in expression (“morph steps”) across continua, right OFA: *t*_(18)_ = 4.29, *p* < 0.0005, left OFA: *t*_(18)_ = 3.61, *p* < 0.005, right FFA: *t*_(18)_ = 4.28, *p* < 0.0005, left FFA: *t*_(18)_ = 4.10, *p* < 0.001. In contrast, linear adaptation effects were not significant in either right or left STS or right or left amygdala (*p*s > 0.1). “amy” = Amygdala. Bars show group mean (±S.E.) One sample *t*-tests against 0, ^**^p < 0.01, ^***^p < 0.001.

A region where we particularly expected to see evidence of graded non-categorical representation of stimuli from the morphed expression continua used here was the OFA. This region is widely thought to support early-stage face processing and to feed low-level feature information forward to other regions that engage in more specialized processing of complex characteristics, such as identity or affect (Haxby et al., 2000; Pitcher et al., 2011a). As such, the OFA was expected to be sensitive to physical differences between sequential faces. In line with this, OFA activity showed a release from adaptation for stimulus changes, both for large changes (across the categorical boundary) and more subtle transitions within faces belonging to a single continuum end. Specifically, results from our categorical model revealed that when expressions at one end of a given continuum followed those from the other end of the continuum, a significant release from adaptation was found: “between end” transitions: right OFA: *t*_(18)_ = 8.21, *p* < 0.0001, left OFA: *t*_(18)_ = 5.94, *p* < 0.0001, Figure 6. Release from adaptation was observed as well for transitions between 50/50 morphs and expressions from either end of a given continuum, “50/50 <> end” transitions: right OFA: *t*_(18)_ = 5.61, *p* < 0.0001, left OFA: *t*_(18)_ = 3.72, *p* < 0.005. Finally, release from adaptation was also observed for small physical transitions within a single end of the expression continua (i.e., between exemplars typically categorized as showing the same expression), “within end” transitions: right OFA: *t*_(18)_ = 4.21, *p* < 0.001, left OFA: *t*_(18)_ = 2.94, *p* < 0.01. The “graded” model further indicated that OFA release from adaptation could be fit by a linear function of physical “morph steps,” across continua, right OFA: *t*_(18)_ = 4.29, *p* < 0.0005, left OFA: *t*_(18)_ = 3.61, *p* < 0.005, Figure 7. Taken together, these findings are consistent with the OFA being sensitive to physical changes in facial expression including those rarely associated with a change in categorical emotion perception. This contrasts with both the amygdala and the superior temporal sulcus (see below), where the graded model did not indicate any significant release from adaptation as a linear function of morph steps between sequential expressions.

An interesting question was whether we would see evidence of graded or categorical representation of emotional expressions within the FFA. Traditionally this region has been implicated in the processing of facial identity as opposed to facial emotion (Haxby et al., 2000) though more recently a number of studies have challenged this perspective (Ganel et al., 2005; Fox et al., 2009; Xu and Biederman, 2010), while others have also questioned the extent to which the FFA may process facial features as well as gestalt representations (Harris and Aguirre, 2010). In the FFA, as in the OFA, release from adaptation was observed, across continua, when expressions at one end of a given continuum followed those from the other end of the continuum, “between end” transitions: right FFA: *t*_(18)_ = 3.85, *p* < 0.005, left FFA: *t*_(18)_ = 3.69, *p* = 0.005, Figure 6. For transitions between 50/50 expressions and expressions from one end of a continuum or the other (“50/50 <> end” transitions), release from adaptation reached significance at *p* < 0.05 in right FFA but not left FFA: *t*_(18)_ = 2.73, *p* < 0.05, *t*_(18)_ = 1.73, *p* = 0.10, respectively. Finally, transitions within a given end of an expression continuum (those unlikely to be associated with a change in categorical emotion perception) were not associated with significant release from adaptation in either right or left FFA, “within end” transitions: *t*_(18)_ = 0.47, *p* > 0.5, *t*_(18)_ = 0.46, *p* > 0.5, respectively. Here, it is of note that “50/50 <> end” adaptation effects were weaker than those observed in OFA and release from adaptation was not observed in FFA, unlike OFA, for “within end” transitions. However, the graded model did indicate that FFA release from adaptation could be fit using a linear function of physical “morph” steps away from one expression toward the other, right FFA: *t*_(18)_ = 4.28, *p* = 0.0004, left FFA: *t*_(18)_ = 4.10, *p* = 0.0007, Figure 6. Release from adaptation for “within end” expression transitions is arguably a more stringent test of the continuous vs. categorical accounts. Taken together, these data suggest that to some extent the FFA seems to fall in-between the OFA and amygdala and STS (see below), showing aspects of both graded and categorical representation of emotional expressions.

The STS has previously been implicated in the processing of facial affect (Harris et al., 2012), especially dynamic facial expressions (Pitcher et al., 2011b; Zhu et al., 2013), and is argued to be responsive to the social relevance of stimuli in the environment. Adaptation effects in the STS, like the amygdala, were not well-characterized by a graded non-categorical model of expression representation; linear adaptation effects for all continua: *p*s > 0.1, Figure 7. Indeed, the categorical model revealed that the STS showed greatest sensitivity to transitions between 50/50 morphs and faces from either end of the continua. Neither right nor left STS showed release from adaptation for “between end” or “within end” transitions, *p*s > 0.1. Across continua, right STS showed release from adaptation for “50/50 <> end” transitions: *t*_(18)_ = 2.13, *p* = 0.047. This however only reached significance within the surprise-fear continuum, right sts: *t*_(18)_ = 2.41, *p* = 0.027. In both the surprise-fear continuum and sad-fear continuum, release from adaptation was significantly greater for “50/50 <> fear” end face transitions than for transitions between 50/50 morphs and non-fear-end (sad or surprise) faces, surprise-fear: left STS, *t*_(18)_ = 2.11, *p* = 0.049, sad-fear: right STS: *t*_(18)_ = 2.44, *p* = 0.025.

##### Effects of anxiety upon representation of emotional expressions across the extended face network

We next looked to see if any region from the extended face network showed an anxiety-related adaptation “bias” for faces from the surprise-fear continuum that paralleled that observed in the right amygdala. This was indeed the case for right FFA. Here, elevated trait anxiety levels were associated with greater adaptation for “50/50 <> fear” end face transitions than for “50/50 <> surprise” end face transitions, *r*_(19)_ = −0.44, *p* = 0.030, one-tailed. (Note, the *r* coefficient is negative here as differential adaptation, i.e., decrease in BOLD, increases with anxiety.)

No equivalent relationship between anxiety and adaptation bias for faces from the surprise-fear continuum was observed in any of our other ROIs of interest. We additionally investigated if there was any other modulation by anxiety of adaptation effects for 50/50 <> end transitions, 50/50 <> end “B” vs. 50/50 <> end “A” transitions (“adaptation bias”), or “between end” transitions for any of the three expression continua across the extended face processing network. No other significant effects were observed within FFA, OFA, or STS. It is also of note that anxiety did not significantly modulate “graded representation” of emotional expression (i.e., adaptation modeled as a linear function of expression morph level) in OFA, FFA, or STS for any of the three continua.

#### Direct effects analyses

The experiment reported here was optimized for investigation of “carry-over” adaptation effects between sequentially presented stimuli. The short inter-stimulus intervals and type 1, index 1 sequence, while ideal for this purpose, provide limited power for detecting a difference in the mean direct effect of one stimulus compared to another. Hence, direct effects analyses are included for completeness, but with this important caveat. Using the categorical model, analyses of covariance (ANCOVA) with Greenhouse-Geisser corrections for violations of sphericity were employed to examine whether there was a main effect of expression type (end “A,” 50/50, end “B”) or an interaction of expression type by anxiety upon the response in the amygdala for each expression continuum separately. In none of the three continua was there either a significant effect of expression type (*p*s > 0.1 except for surprise-fear where *p* > 0.05 for the right amygdala) or a significant interaction of expression type by anxiety (*p*s > 0.1). *Post-hoc* tests applied to surprise-fear continuum activation in the right amygdala revealed a non-significant trend for 50/50 surprise/fear morphs to elicit higher amygdala activity than surprise end or fear end faces (*p* = 0.176, *p* = 0.153 bonferonni corrected, respectively). Using the linear model, the only significant finding was for the surprise-sad continuum, where a significant increase in amygdala activity was observed as a function of increased percentage of sadness in the expression, left amygdala: *t*_(18)_ = 2.57, *p* = 0.019, right amygdala: *t*_(18)_ = 2.31, *p* = 0.033. For the linear model analyses of direct effects, as for the categorical model analyses, no significant interactions of expression level by anxiety were observed.

Parallel “direct effects” investigations of effects of anxiety, expression and their interaction for the three continua were conducted for the extended face network ROIs (OFA, FFA, STS). Here, again, no significant interactions of anxiety by expression were observed using either the categorical or linear model. The only significant effect of expression level was observed within the surprise-sad continuum. Here, the graded model revealed that bilateral OFA activity increased as a function of increasing sadness in the expression, left, *t*_(18)_ = 2.79, *p* = 0.012, right, *t*_(18)_ = 2.12, *p* = 0.048, paralleling our findings for the amygdala. In addition, the categorical model revealed a significant main effect of expression type in right FFA for the surprise-sad continuum, *F*_(2, 34)_ = 4.39, *p* = 0.023. Here, there was no support for a linear increase in activity as a function of increasing sadness. *Post-hoc* tests revealed a significant difference in activity between sad expressions and 50/50 sad/surprise morphs, rFFA activity being higher for the former (*p* = 0.020, bonferonni corrected).

## Discussion

The primary purpose of this study was to investigate anxiety-related biases in the neural representation of facial expressions within the amygdala. Three expression continua were considered. Two of these contained expression blends generated by morphing between a face showing fear and the same identity showing either sadness or surprise. The third continuum was created by morphing between expressions of surprise and sadness, and hence did not involve any percentage of fear. Our aim was to determine whether elevated trait anxiety would be associated with biases in representation in the amygdala of expressions containing some element of fear, and whether this would parallel biases in the perceptual categorization of these expressions.

It has previously been reported that high trait anxious individuals show an increased propensity to categorize expressions mid-way between fear and either surprise or sadness as showing fear (Richards et al., 2002). In the current study, we considered surprise-fear and sad-fear expression continua separately and replicated Richards and colleagues findings of an anxiety-related bias in categorization performance for the former but not the latter continuum. Specifically, for the surprise-fear continuum, individuals with elevated trait anxiety showed a more rapid transition from categorizing expressions as surprise to categorizing them as fear, as a function of the percentage of fear in the morph presented. This led to increased fear categorizations for morphs in the middle of the surprise-fear continuum. There were no significant anxiety-related biases in categorization performance for the sadness-fear or surprise-sadness continua.

The specificity to the surprise-fear continuum of the anxiety-related bias in expression categorization was paralleled by findings from analyses of BOLD adaptation within the amygdala. In line with our a priori hypotheses, trait anxiety modulated the extent of adaptation observed in the amygdala for transitions between faces with 50/50 surprise/fear expression blends and faces showing expressions from the fear vs. the surprise end of the surprise-fear continuum. High trait anxious individuals showed greater adaptation for transitions between 50/50 surprise/fear morphs and faces from the “fear” end of the continuum, while low trait anxious individuals showed the reverse pattern with adaptation being greater for transitions between 50/50 morphs and faces from the “surprise” end. If fMRI adaptation is held to provide an index of representational similarity within a brain region (Grill-Spector et al., 2006), then we may infer that elevated trait anxiety is associated with increased similarity in the amygdala encoding of 50/50 surprise-fear blends and expressions primarily categorized as showing fear.

As was the case for categorization behavior, no anxiety-related biases in amygdala adaptation were observed for expressions comprising blends of sadness and fear, or indeed those comprising blends of surprise and sadness. This suggests that anxiety-related bias in the representation of facial expressions is specific to the surprise-fear continuum, out of the three continua considered, at least for the stimuli used here. Replication of this study with alternate face stimuli or a wider set of facial identities would be of value in establishing the generalizability of this finding. The expressions of fear and surprise are more commonly mistaken for each other than expressions of sadness and fear (Young et al., 1997). Hence, one possibility is that an anxiety-related “fear” bias is only apparent when expressions are easily confusable. In future work, restriction of the stimuli used to faces close to the normative categorization boundary for each continuum might enable us to examine this possibility further, and to determine if an anxiety-related bias is seen in the sad-fear continuum when a smaller range of expression variation is used.

Elsewhere, it has been argued that surprise expressions are themselves ambiguous in indicating a positive or negative event (Kim et al., 2003). This raises the alternate possibility that high trait anxious individuals are more sensitive to negative interpretations when fear is blended with surprise than low anxious individuals but that this bias does not emerge when both ends of the continuum are uniformly perceived as unambiguously negative. It is also interesting to note that neither anxiety-related biases in amygdala activity nor categorization behavior were observed when surprise was present in the context of sadness. The interpretation of surprise as indicative of threat may be constrained by the other emotional elements present in a given expression (see Neta et al., 2011 for further discussion of contextual influences on processing of expressions).

We also examined adaptation effects in three other regions widely held to be core components of a right lateralized face processing network—namely OFA, FFA, and STS. Release from adaptation within OFA was consistent with suggestions that this region may be engaged in fairly early processing of physical face features (Haxby et al., 2000). Specifically, OFA activity increased linearly as a function of change in expression “morph steps” between sequentially presented faces. In addition, release from adaptation in this region was not only observed for faces from different continuum ends, but also for faces from the same end of a given continuum. This hence fits better with graded than categorical representation of expressions (or their component features) within this region.

In line with recent findings (Fox et al., 2009; Xu and Biederman, 2010), FFA also showed release from adaptation as a function of changes in expression, across all three continua. This is contrary to early models suggesting a specialized role for the FFA in processing invariant aspects of facial information such as identity (Haxby et al., 2000). One possibility is that processing of facial features might not be the province of the OFA alone but might entail reciprocal interactions between the OFA and FFA (Rossion, 2008). This however does not explain why the FFA showed less sensitivity to within continuum end changes in expression than OFA, suggesting that it is more sensitive to expression gestalts and less to specific physical changes in component features. This might perhaps be explained by the FFA acting as a core hub for face processing, receiving input from both the OFA and amygdala. Specifically, the FFA might be responsive to feed-forward information about differences in facial features from OFA but also more categorical information about facial expression, especially when threat-relevant, from the amygdala. This would be consistent with prior suggestions that amygdala activity may influence activity in the FFA, modulating its response to fearful faces (Vuilleumier et al., 2001), and could potentially also explain the finding that right FFA adaptation for transitions between 50/50 surprise-fear morphs and “fear end” vs. “surprise end” faces showed parallel modulation by anxiety to that observed in the right amygdala.

We also examined adaptation within the superior temporal sulcus. Here recent studies have reported somewhat contradictory findings. While some studies have reported adaptation in the STS BOLD response as a function of changes in facial expression (Winston et al., 2004), others have reported a failure to find such adaptation effects (Cohen Kadosh et al., 2010; Xu and Biederman, 2010). It has been argued that this might potentially arise from the STS being predominantly responsive to dynamic faces including, under some circumstances, perceived motion between sequential static expressions. Findings from our current experiment provide some support for STS encoding of facial expression. As in the amygdala, the pattern of adaptation observed was better fit by a categorical than a continuous model of expression representation. Also sharing similarities with findings for the amygdala, across subjects, STS adaptation was greater for 50/50 expression morphs followed or preceded by expressions from the non-fear end of the surprise-fear and sad-fear continua. This potentially suggests differential representation of faces with 67% or higher fear content, perhaps reflecting the social relevance of such expressions as cues of potential threat in the environment. Interestingly, this adaptation bias was superimposed on a general tendency for release from adaptation in the STS for transitions between 50/50 expression morphs and faces from either continuum end. One possibility is that this could reflect the STS having a general role in resolving the meaning of expressions. This might lead to greatest sensitivity to transitions to and from 50/50 expressions, and also account for sensitivity to dynamic expressions, which by default involve a change in meaning. An alternative, though less likely, possibility is that these 50/50 <> end transitions may be the most able to create a percept of dynamic expression, with continuum end to end transitions potentially being too large to create such a percept.

To conclude, our current findings suggest that trait anxiety is not only associated with threat-related biases in the categorization of surprise-fear expression blends, but also with altered representation of these stimuli in the amygdala. Specifically, trait anxiety levels significantly correlated with the extent to which participants showed greater adaptation of the amygdala BOLD response, and hence arguably greater representational similarity, for transitions involving 50/50 surprise/fear morphs and faces from the fear, vs. surprise, end of the continuum. This finding was only observed when fitting a categorical model and not a graded, linear, model to the amygdala BOLD signal. A similar pattern was also observed in the right FFA. This latter region, however, was also sensitive to graded physical changes between facial expressions, though to a lesser extent than the OFA. These findings provide initial evidence as to the neural correlates of biases in face expression perception shown by high trait anxious individuals.

### Conflict of interest statement

The authors declare that the research was conducted in the absence of any commercial or financial relationships that could be construed as a potential conflict of interest.
