# Supplementary figures and images for: Seeing the world through non rose-colored glasses: anxiety and the amygdala response to blended expressions
Source: Front Hum Neurosci. 2015 Mar 27;9:152. doi: 10.3389/fnhum.2015.00152 (PMC4375986; doi:10.3389/fnhum.2015.00152)

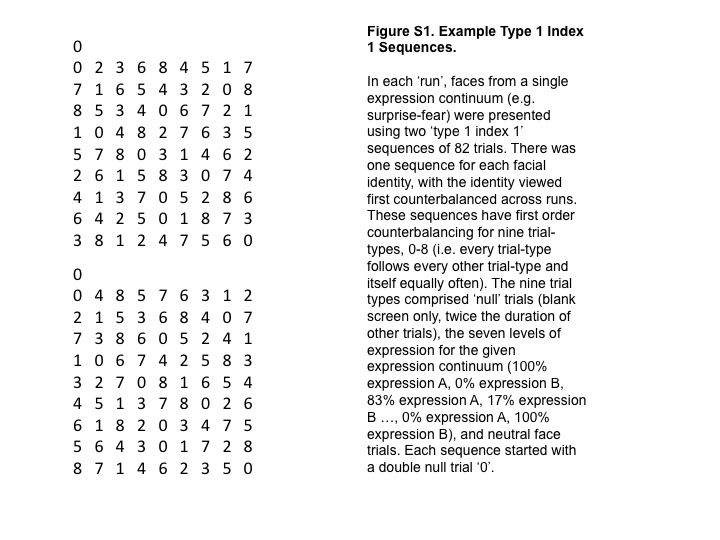

Supplement: Supplementary file 1 [file SupplementaryFigure1.JPG]

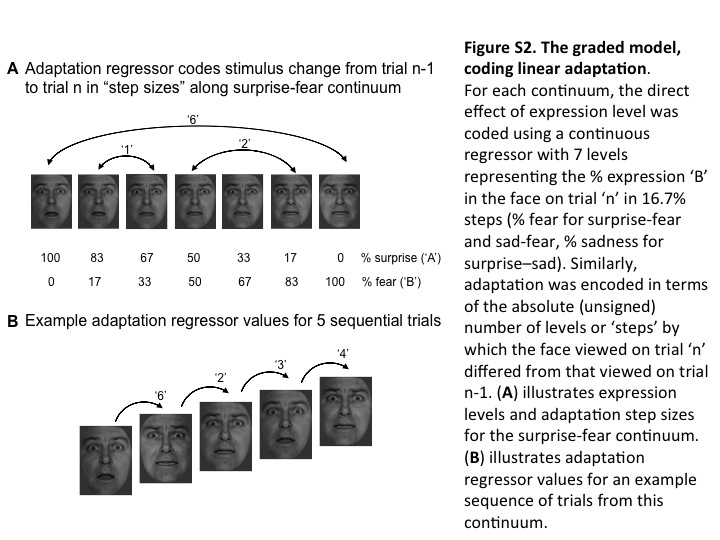

Supplement: Supplementary file 2 [file SupplementaryFigure2.JPG]
